# The Phenox Clot Retriever as Part of a Multimodal Mechanical Thrombectomy Approach in Acute Ischemic Stroke: Single Center Experience in 56 Patients

**DOI:** 10.1100/2012/190763

**Published:** 2012-04-24

**Authors:** Sascha Prothmann, Hannah Lockau, Franziska Dorn, Holger Poppert, Annette Förschler, Claus Zimmer, Thomas Liebig

**Affiliations:** ^1^Department of Neuroradiology, Klinikum rechts der Isar, Technische Universität München, Ismaninger Straße 22, 81675 Munich, Germany; ^2^Institute and Policlinic of Radiological Diagnostics, Uniklinik Köln, Kerpener Straße 62, 50937 Cologne, Germany; ^3^Neurological Clinic and Policlinic, Klinikum rechts der Isar, Technische Universität München, Ismaninger Straße 22, 81675 Munich, Germany

## Abstract

*Purpose*. We analyzed our experience with the phenox clot retriever as part of a multimodal mechanical thrombectomy (MTE) approach in acute ischemic stroke. *Methods*. 56 patients were treated by MTE with the phenox clot retriever alone or in combination with other modalities. *Results*. Overall we achieved TICI 2b/3 reperfusion rates of 61,9%. In multimodally treated patients we achieved reperfusion rates of 72,8%. There were 3 (5,5%) severe adverse events, all symptomatic intracranial hemorrhages. The mean angio to reperfusion times (ART) were 74 minutes for phenox-only procedures and 51 minutes for multimodal procedures. A chronological analysis showed a reduction of ART from 70,5 to 49,4 minutes and an increase of TICI 2b/3 recanalizations from 53,8% to 81,8%. Throughout the observation period there was a significant shift towards multimodal procedures with simultaneous increase of TICI 2b/3 reperfusions. Both effects are partially attributable to our institutional learning curve. NIHSS improvement could be seen in 54% (*n* = 28) overall and in 73% (*n* = 15) of MCA recanalizations. *Conclusions*. The phenox clot retriever is a safe and effective tool for MTE in acute stroke patients, with faster and better reperfusion results when used as part of a multimodal strategy. Clinical improvement is more frequent in MCA recanalizations.

## 1. Introduction

After the large intravenous thrombolysis trials have shown that the effectiveness of IV rTPA is limited especially in severe stroke due to large vessel occlusion, mechanical thrombectomy (MTE) has become the focus of a number of trials leading to the approval of dedicated thrombectomy systems such as the Merci retriever and the Penumbra aspiration thrombectomy system [1–3]. In addition to FDA-approved systems, a number of MTE systems have become available under CE mark over the past 3 years. One of these systems is the phenox clot retriever (phenox GmbH, Bochum, Germany). Licensed under CE mark in November, 2006, it essentially resembles a microbrush that is delivered and released through a microcatheter and then withdrawn into an aspiration catheter—either guiding or distal access. Available in three different sizes, it can be used in vessel diameters between 1 and 6 mm. By design, it offers the unique possibility to use two devices simultaneously, for example, in cases of a bifurcation occlusion. In this retrospective study, we analyzed our experience with the phenox clot retriever as part of a multimodal thrombectomy approach in acute ischemic stroke.

## 2. Material and Methods

56 patients with acute ischemic stroke in the anterior and/or posterior circulation were treated with the Phenox clot retriever as the sole device or as part of a multimodality strategy in combination with other devices between 01/2007 and 08/2010. Treatment was initiated within 6 hours of symptom onset for the anterior circulation and 8 hours for the posterior circulation. Neurological evaluation (NIHSS, mRS) was performed on admission and at discharge. All patients underwent CT and CTA on admission to rule out hemorrhage and to evaluate vessel occlusion. Unenhanced CT was repeated 18 h later (±6 h) and at discharge.

According to institutional guidelines, all patients who presented with symptom onset ≤3 h due to large artery occlusion received IV rtPA immediately after the CT scan while being transferred to our angiography suite for subsequent MTE. All except one procedures were performed under general anaesthesia and were carried out by a constant team of three neuroradiologists. IV lysis was stopped when the guiding catheter was positioned. If necessary, the remaining dose, up to a maximum of 0,9 mg/kg bodyweight and a total of 90 mg, was administered i.a. via a microcatheter. In case of contraindications against IV lysis or symptom onset ≥3 h, MTE was performed without adjunctive fibrinolysis. For the analysis, all patients treated with the phenox clot retriever alone or in combination with another thrombectomy/aspiration devices and/or ia lytics were selected from our institutional database of more than 180 endovascular treatments of ischemic stroke.

**Figure 1:**
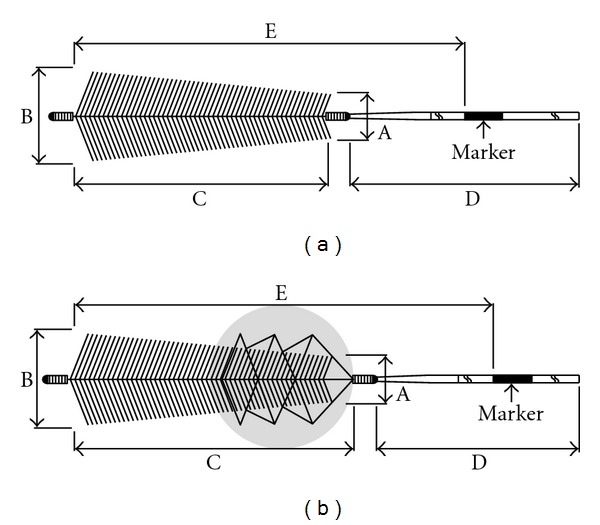
Schematic drawing of the phenox clot retriever ((a) pCR) and the phenox clot retriever cage ((b) CRC).

The Phenox Clot Retriever (pCR, phenox, Bochum, Germany) consists of a flexible nitinol/platinum core wire surrounded by perpendicularly oriented polyamide microfilaments. These form a conical shape with increasing diameter towards the distal end covering 3 different sizes ranging from 1 to 3 mm proximally and from 2 to 5 mm distally. The smallest is 10 mm in length and is introduced through a 0.021′′ microcatheter, while the two larger versions are 20 mm in length and require a 0.027′′ catheter (Figure 1). The 2/4 mm and 3/5 mm are also available with a nitinol cage attached to the proximal marker that should help to centralize the device within the vessel and also allow for enhanced thrombectomy (CRC, clot retriever cage) (Figure 1). All phenox retrievers should be deployed distal to the thrombus and then slowly withdrawn under constant aspirating on the guiding catheter by means of a 50 cc syringe.

Because of their backwards-oriented filaments that make resheathing impossible, both pCR and CRC retrievers are single use. Therefore, the maximum number of unsuccessful attempts with the first type of device was three. Thereafter, we pursued an escalative strategy with a different device. Besides pCR and CRC, we used the Merci retriever (L series), and the Penumbra aspiration system, percutaneous transluminal angioplasty (PTA) with the Ryujin balloon catheter, permanent or temporary stenting with the Solitairestent and the Enterprisestent, the Catch system, and a microsnare. The order of devices within multimodal treatment varied.

Recanalization and reperfusion success were rated on pre- and postprocedural angiograms according to the Thrombolysis in Myocardial/Cerebral Infarction (TIMI and TICI) grading system [4]. TICI IIb and III were accounted for as a potential beneficial reperfusion results. Times measured and documented included symptom onset to groin puncture (time to angio), onset to first reperfusion of TICI 2a or better (time to reperfusion) groin puncture to first reperfusion (angio to reperfusion), and groin puncture until the end of the procedure.

Clinically significant procedural adverse events (PAE) and symptomatic intracranial haemorrhage (sICH) were defined by a CT scan within 24 h posttreatment and an increase on the NIHSS of 4 or more points. Clinical improvement/deterioration regarding the early outcome was defined as a pre-/post-NIHSS difference of 4 or more points and any difference of the mRS score.

Statistical analysis included descriptive statistics. Tests for correlation and for significance of differences were performed by using SPSS.

## 3. Results

56 patients (33 male/23 female) with a mean age of 69 years (min. 41, max. 93) presented with a mean NIHSS score of 16,6. The site of occlusion was internal carotid artery (ICA)/Carotid T in 17/56 (30%), middle cerebral artery (MCA) in 25/56 (45%), and basilar artery (BA) in 14/56 (25%) cases. 33 (59%) procedures were performed with pCR or CRC as the only MTE device. The remaining 23 (41%) procedures were performed with the pCR/CRC in combination with one or more of the following devices: Penumbra system (8/23), the Solitairestent (12/23), Enterprisestent (1/23), the Merci L5 (3/23), the Catch device (2/23), and a microsnare (3/23).

**Figure 2:**
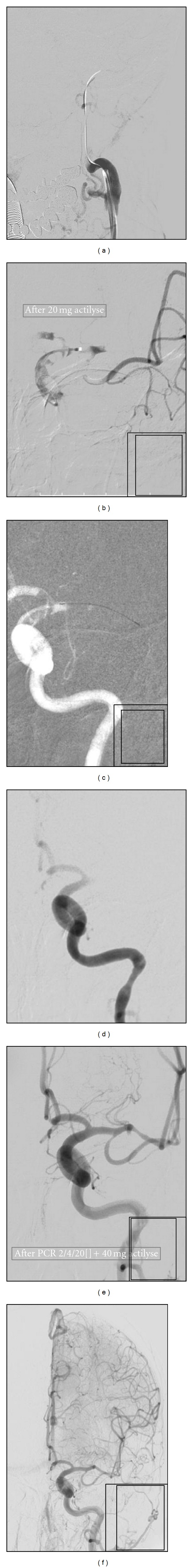
(a) Acute left-sided occlusion of the carotid T; (b) partial recanalization after administration of 20 mg intraarterial rtPA; (c) placement of the pCR 2/4/20 (arrows) in the ACM (M1) distal to the thrombus; (d) recanalization of the carotid T and ACA after retraction of the pCR with thrombus left in the M1 segment; (e) and (f) TICI 2a (TIMI 2) reperfusion after second passage with the pCR and administration of 40 mg intraarterial rtPA collectively. The inferior M2 branch remains occluded.

4 patients were treated with IV tPA only (min. 50 mg, max. 72 mg) before carrying out mechanical thrombectomy (MTE). In 16 patients, MTE was preceded by intraarterial tPA (min. 5 mg, max. 40 mg), and 17 patients received both intravenous and intraarterial tPA (min. 21 mg, max. 97 mg) (Figure 2). 19 patients were treated with MTE without thrombolysis. Statistically, no significant effect on recanalization rates nor an increased rate of hemorrhage in connection with lytics could be observed.

Overall, we achieved a potentially beneficial TICI IIb/III reperfusion rate of 61,9% and a TIMI II/III reperfusion rate of 85,5% (Figure 5). Regarding the unimodal procedures with pCR/CRC alone (*n* = 33), the TICI IIb/III recanalisation was 54,5% and 84,2% TIMI II/III, respectively, whereas the multimodal procedures (*n* = 22) could even yield a 72,8% TICI IIb/III and 86,4% TIMI II/III reperfusion. However, this difference did not reach a level of statistical significance.

The mean angio-to-reperfusion times (ART) were 74 minutes for pCR/CRC-only procedures (SD ± 54) and 51 minutes for multimodal procedures (SD ± 29) in average. This difference is significant on a 10% level (*P* = 0.065, Mann-Whitney *U*) (Figure 6). A chronologic analysis of ART also showed a reduction over time from an average of 70,5 min (SD ± 30,4) for the first 10 procedures to 49,4 (SD ± 26,7) for the last 10 (Figure 7). Likewise, we observed a significant shift from mono- to multimodal procedures during the observation period from 25% for first 12 cases to 91,7% for last 12 cases (*P* = 0,001, *χ* ^2^) with a simultaneous increase of potentially beneficial reperfusion results (TICI IIb/III) from 53,8% to 81,8% referring to the first and last 12 procedures.

**Figure 3:**
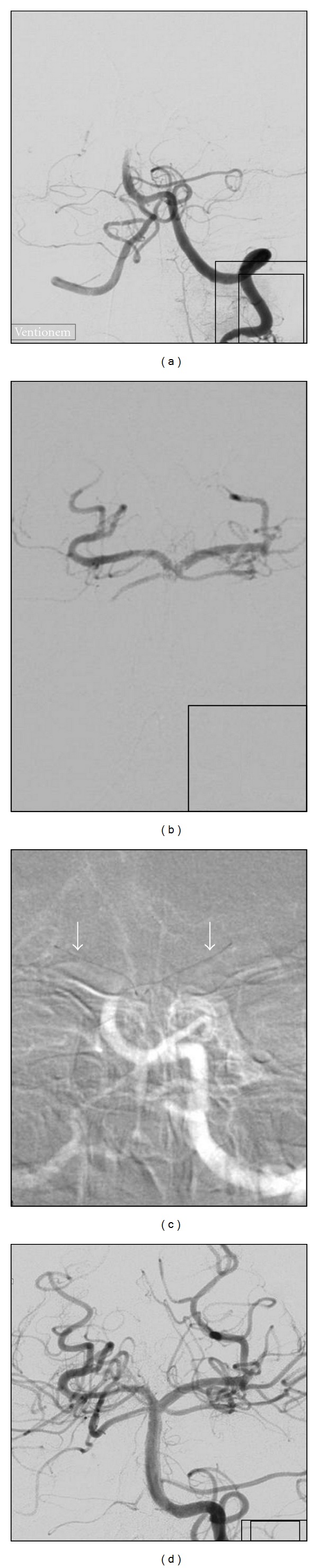
(a) Acute distal basilar artery occlusion, application of 10 mg IA rtPA without visible effect; (b) passage of the thrombus with guidewire and microcatheter, tip of the basilar artery and both posterior cerebral arteries are open; (c) placement of pCR 2/4/20 in each P1-segment of the posterior cerebral artery (arrows); (d) simultaneous retraction of the thrombectomy device into the guiding catheter leads immediately to full (TICI 3) reperfusion (kissing technique).

**Figure 4:**
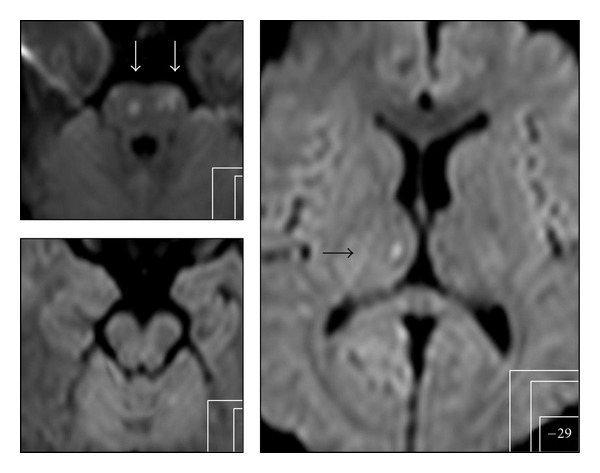
Very few DWI lesions in follow-up MRI 24 h after symptom onset in pons (white arrows) and in the right thalamus (black arrow) and no neurological deficit.

**Figure 5:**
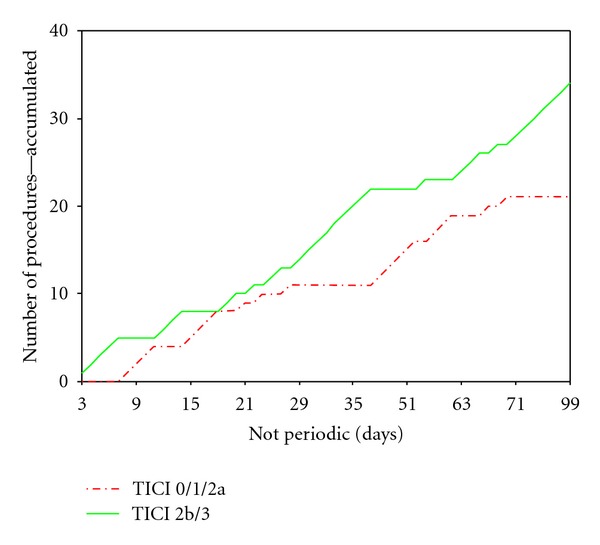
The diverging graphs demonstrate the increasing preponderance of potentially beneficial TICI 2b/3 reperfusion results (green line) over the frustrating recanalization results (TICI 0/1/2a, red line) in the time course of the observation period. This observation is partially attributable to our institutional learning curve.

**Figure 6:**
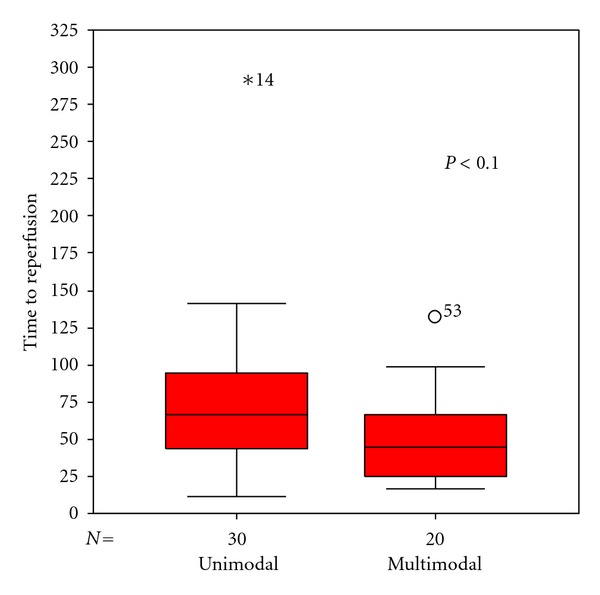
The box plot shows the significant (*P* < 0.065, Mann-Whitney *U*) shorter angio-to-reperfusion times of multimodal procedures compared with unimodal procedures.

**Figure 7:**
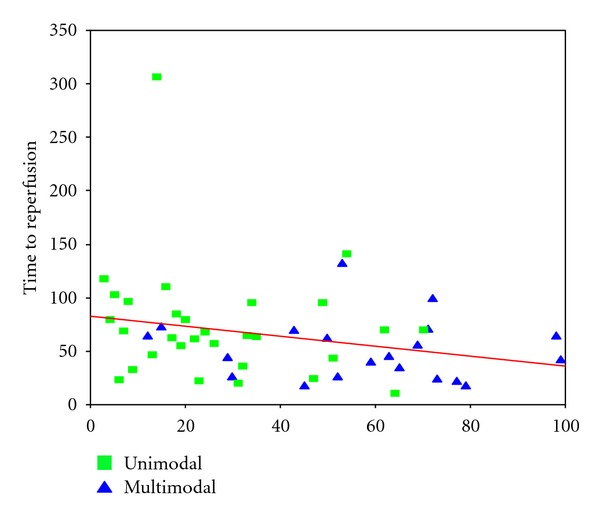
The scatter plot visualizes 2 interesting observations. Throughout the observation period, we used increasingly a multimodal recanalization strategy with simultaneously decreasing reperfusion times.

Clinical improvement after the procedure could be seen in 54% when evaluated with NIHSS (*n* = 28) and in 37% according to the mRS (*n* = 38). NIHSS improvement was largely dependent on the vascular territory. It occurred in 73% of MCA recanalizations (*n* = 15) and in 57,1% of ICA occlusions (*n* = 7). Only one patient improved after BA recanalization (Figures 3 and 4).

30,4% of the patients (17/56) died during their hospital stay. These patients had an initial mean NIHSS of 22 (SD ± 12), an ICA/Carotid-T occlusion in 5, an MCA occlusion in 6, and an occlusion of the BA in 6 cases. The mean time to first reperfusion for these patients was 323 minutes; the reperfusion result was TICI IIb/III in 10/17 cases and TICI 0-IIa in 7/17 cases.

There were 3 procedural adverse events, each without clinical consequences referred to a change in NIHSS ≥4 and no device-related adverse event. The rate of SICH after treatment was 5,5% (3/56).

## 4. Discussion

Since the first promising case reports and in vitro study in 2006 and 2008 [5, 6], this is the first study that demonstrates the feasibility and effectiveness of the phenox system alone or as a part of a multimodality approach to MTE under clinical conditions in acute stroke patients.

For the evaluation of our reperfusion results, we decided to use the more refined TICI classification (instead of TIMI) because of the graduation of the category “partial recanalization” (TICI II) in less or more than 2/3rds reperfusion of the dependent territory. This differentiation seemed to be reasonable with respect to expected patient outcome. Based on this consideration, we suggest that reperfusion of less than 66% of the dependent territory (TICI IIa) is substantially less beneficial than ≥66% reperfusion (TICI IIb/III). Hence, successfule recanalization was defined as a TICI IIb/III result. However, most likely due to the heterogenity of our sample and probably due to some lacking outcome data, no statistical proof for this assumption could be found in this retrospective study. Nevertheless, many other studies have already proven that recanalization of the occluded vessel is associated with better outcome [1–3, 7].

Regarding our sample, especially the patients with MCA occlusions took advantage of MTE, followed by the patients with ICA occlusions. No statistically proven clinical benefit was seen in patients with BA occlusions. One reason could be collateral flow, probably more often present in MCA occlusions than in ICA or BA occlusions. Anyhow, when looking at the poor outcome data of the BA occlusions, one should keep in mind that according to previous studies the percentage of patients that actually died or showed clinical worsening was much lower than the natural history of this disease [8].

There are some issues when comparing our reperfusion results with other mechanical thrombectomy studies, mostly because of the different definitions of a successful recanalization and reperfusion. The Multi Merci Trial has the most comparable definition with similar reperfusion results [1]. Seemingly better reperfusion results are reported for the Penumbra system [3, 9–11], whereas in these studies a TIMI II result in the target vessel is already valued as a successful recanalization. This might be one cause for the relative lower rate of good clinical outcome (25%) despite the higher recanalization rate of 81,6% in the penumbra pivotal stroke study [3].

In the present study, mechanical thrombectomy with the pCR/CRC yielded TICI IIb/III recanalization in 54,5% (*n* = 33) when used unimodally and up to 72,8% (*n* = 22) when applied in a multimodal manner. Even though this effect did not reach a level of significance, it is remarkable because of a similar observation made in the Multi Merci Trial with the newer generation L5 Merci retriever [1]. In this study, TIMI II/III reperfusion was achieved in 57,3% when the procedure was done with the Merci retriever exclusively, whereas 69,5% could be reached when the retriever was combined with intraarterial tPA or other mechanical devices.

Another positive effect of the multimodal approach could be found regarding the procedure times. ART decreased significantly when we pursued an escalative strategy with rapid changing of the device after a limited number of unsuccessful attempts. Another interesting fact is the trend towards shorter ARTs and a shift of the treatment strategy from uni- to multimodal in the time course of the observation period. Thus, the reason for shorter procedure times at the end of the observation period can be both increasing experience on one hand and the use of a more successful multimodal recanalization strategy on the other hand.

We had an acceptable low percentage of symptomatic intracranial hemorrhages (SICH) of 5,5% (3/56) which is substantially lower than in the PROACT II study and the Multi Merci Trial where SICH occurred in 10,9% and in 9,8%, respectively [1, 7, 12]. Besides, there was no device-related complication.

## 5. Conclusion

The phenox clot retriever pCR and CRC have proven to be safe and effective tools for mechanical thrombectomy in acute stroke patients. When used exclusively in a unimodal manner, successful recanalization could be reached in 54,5% opposed to 72,8% in the multimodality setting. The escalative strategy showed two major advantages: a higher percentage of TICI IIb/III reperfusion rates and shorter angio-to-reperfusion times. However, these effects are at least partially attributable to our institutional learning curve. Clinical improvement is more frequent in MCA recanalizations than in BA recanalizations. Despite our escalative strategy, no increase in the rate of sICH over previous studies and no adverse events due to the phenox clot retriever could be observed.
